# Design, Development and Validation of a Training Package for Treating Procrastination in Students Based on Grounded Theory: A Mixed‐Methods Approach

**DOI:** 10.1002/pchj.70114

**Published:** 2026-07-01

**Authors:** Sadegh Hekmatiyan Fard, Seyed Mousa Golestaneh, Mahnaz Joukar Kamal Abadi, Yousef Dehghani

**Affiliations:** ^1^ Department of Psychology, Faculty of Literature and Humanities Persian Gulf University Bushehr Iran

**Keywords:** academic procrastination, mixed‐methods research, procrastination treatment package, university students

## Abstract

The present study aimed to design, develop, and validate a training package for treating student procrastination based on grounded theory. In the first phase, a qualitative grounded theory study was conducted to develop this intervention. The study population consisted of students enrolled at the Persian Gulf University in the academic year 2023–2024. In the quantitative phase of this study, a quasi‐experimental pre‐test‐post‐test control group design was employed. A multi‐stage random sampling technique was used to select participants. The procrastination questionnaire was randomly distributed to 1000 students from the Persian Gulf University, stratified by faculty, major, and year of entry. The findings indicated that the procrastination treatment package was effective in reducing procrastination among students.

## 1 Introduction

College life involves a seemingly endless series of challenging tasks, providing students little respite. However, it is not the great number of tasks that troubles students, but their tendency to delay the tasks, in comparison to their goals (Sun and Kim 2023). Henceforth, the challenges arise from the student's inability to self‐regulate, stay dedicated, and finish the tasks. Procrastination refers to the delaying of tasks (Zhang et al. 2023). In other words, procrastination is the postponement of work without a specific cause (Turner and Hodis 2023).

Approximately a quarter of students report that procrastination causes tension for them and disrupts their academic performance. This hinders their ability to utilize their full potential in their learning process, leading to eventual failure (Ren et al. 2023). Research shows that procrastination harms health and well‐being, and life satisfaction among different populations (Narci 2022). Intrapsychic outcomes of procrastination include psychological distress, self‐criticism, hopelessness, and regret. External outcomes of procrastination can be significant and detrimental to the student's academic performance. For example, it can cause individuals to miss opportunities and harm their relationships. It can also negatively predict academic grade averages and, consequently, academic probation (Caratiquit and Caratiquit 2023), causing a financial burden on the student.

Research points to various antecedents of procrastination. A common belief about the nature of procrastination is that it stems from high standards (Kurtovic et al. 2019). A tendency to attain perfect and unattainable standards is characteristic of perfectionist beliefs (Stoeber et al. 2020). Delaying a task can be due to a tendency to strive for perfection, which can make completing tasks tedious or unpleasant. Therefore, in the face of perfectionist standards, procrastinators may deem it too early to address a task and consequently evade completing it. Procrastination might be the consequence of factors such as low self‐efficacy and irrational beliefs (Ma et al. 2023). Moreover, procrastination can be a detrimental habit with consequences such as stress, worry, fear of failure (Cho and Lee 2022), constant anxiety, and depression (Kınık and Odacı 2020).

As noted, procrastination can affect various aspects of individual and social life, education and academic performance, finances and subsequently mental and physical health. It can also manifest in a person's career, affecting organizations (Barabanshchikova et al. 2018). Research in this field can help design measures and interventions to reduce or eliminate procrastination. Academic procrastination is prevalent among students, is on the rise, and has unpleasant consequences. It is related to multiple factors, and research findings vary among students of different majors, genders, and ages. Considering the mental health implications, preventing academic procrastination, and empowering students are essential. Academic procrastination causes various issues in different educational settings, leading to challenges, misbehaviors, and problems. Henceforth, this study could have significant implications for the academic community. This research aims to study procrastination from the perspective of students who experience it.

Despite extensive evidence on the correlates and consequences of academic procrastination, there remains a practical gap in developing interventions that are explicitly derived from students' lived experiences and translated into a coherent, theory‐driven treatment protocol. In many contexts, intervention programs are adopted from existing models without being systematically grounded in the target population's explanatory processes and coping patterns.

Therefore, the present study addresses this gap by (1) generating a grounded, data‐driven explanatory framework of how procrastination forms and is maintained among university students at Persian Gulf University, (2) translating the resulting categories and core process into a structured training/treatment package, and (3) empirically testing the effectiveness of this package in reducing students' procrastination using a quasi‐experimental pre‐test/post‐test control‐group design.

Our research questions and hypothesis were as follows:

Research Question 1 What causal factors influence the formation of procrastination?

Research Question 2 What contextual and mediating factors influence the formation of procrastination?

Research Question 3 What actions and strategies are employed to address procrastination?

Research Question 4 What are the consequences of procrastination?

Research Question 5 What is the core phenomenon underlying the formation of procrastination?

Hypothesis 1 *The educational package for the treatment of procrastination has a significant effect on the procrastination of students*.

## 2 Method

### 2.1 Participants and Methodology

This study employed a mixed‐methods approach. The qualitative phase utilized Grounded Theory methodology, focusing on the systematic collection and analysis of data to develop a theory grounded in the empirical observations. The rationale for using a mixed‐methods approach stems from the complexity of procrastination as a phenomenon, which involves both subjective experiences (best explored qualitatively) and measurable behavioral changes (suited for quantitative analysis). By combining these methods, we aimed to ensure that our theoretical findings could be grounded in real‐life experiences and subsequently validated through a structured intervention.

The population for the qualitative phase consisted of students enrolled at Persian Gulf University during the academic year 2023–2024. Initially, the Tuckman Procrastination Questionnaire was administered to 1000 volunteer students. Students who scored two standard deviations above the mean (procrastinators) were selected for the qualitative sample, comprising 22 undergraduate students (17 female, 5 male). The selection process was purposive, aiming to capture a range of experiences related to procrastination.

From the pool of 1000 volunteer students who completed the Tuckman Procrastination Questionnaire, individuals scoring at least two standard deviations above the sample mean were identified as eligible for the qualitative phase. Eligible students were approached and invited to participate in semi‐structured interviews; those who provided written informed consent were enrolled. Sampling for the interviews began as purposive (targeting high scorers) and then proceeded as theoretical sampling: based on the preliminary codes from each interview, subsequent participants were selected from the remaining eligible pool to further elaborate emerging categories and to seek variation in experiences relevant to underdeveloped concepts. Interviews continued until theoretical saturation was achieved, resulting in 22 undergraduate interviewees.

### 2.2 Interview Guide Development

The semi‐structured interview guide was developed in an iterative, theory‐informed manner consistent with grounded theory methodology. First, an initial pool of interview questions was generated based on (a) the study aims and research questions (RQ1–RQ5), and (b) a focused review of prior empirical and theoretical work on academic procrastination (e.g., antecedents, maintaining mechanisms, coping strategies, and consequences). The initial questions were mapped onto the grounded‐theory elements of conditions, actions/interaction strategies, and consequences, to ensure alignment with the intended analytic framework.

Second, the draft guide was reviewed by 6 experts in psychology/qualitative research to evaluate clarity, relevance, and sensitivity of the wording. Based on their feedback, redundant or leading questions were removed, and prompts were refined to encourage narrative depth and concrete examples.

Third, pilot interviews (*n* = 5) were conducted with eligible students to assess comprehensibility, flow, and interview length. Feedback from the pilots led to revisions in question order, the addition of probes (e.g., examples of typical delayed tasks, emotional reactions, and contextual triggers), and simplification of ambiguous terms.

Finally, consistent with constant comparative analysis and theoretical sampling, the interview guide remained flexible: after each interview, emerging codes informed minor revisions (e.g., adding follow‐up probes to explore underdeveloped categories) while retaining core questions for comparability across participants. The final interview protocol is provided in Appendix A.

### 2.3 Data Collection and Grounded Theory Stages

Semi‐structured interviews, lasting between 45 and 90 min, were conducted at a time convenient for participants. All interviews were recorded, transcribed, and analyzed iteratively using Constant Comparative Analysis, a key process in Grounded Theory. Following each interview, initial transcripts were reviewed, and emergent themes or codes were identified, which informed subsequent interviews (theoretical sampling). This allowed the research team to refine the focus of the interviews and explore emerging concepts in greater depth.

Data collection continued until theoretical saturation was reached—when no new significant categories or insights emerged from the interviews. Throughout this process, open coding was employed to break down the data into discrete parts, examining them for similarities and differences. These codes were constantly compared with each other to develop preliminary categories.

### 2.4 Axial Coding and Data Categorization

Once open coding was completed, the research moved into the phase of axial coding, where the relationships between categories were examined and organized into more refined categories based on their properties and dimensions. This stage involved exploring the conditions that led to procrastination, its context, intervening factors, and the consequences of these behaviors as expressed by participants. The process of axial coding helped the researchers identify the core category around which all other categories were linked.

### 2.5 Selective Coding and Theory Development

Finally, the selective coding phase began, focusing on integrating and refining the categories into a cohesive theoretical framework. The core category identified—procrastination as a coping mechanism to manage academic pressure—was explored in relation to its sub‐categories, such as emotional regulation, time management, and personal motivation. The team used memo writing throughout the analysis to document insights, emerging patterns, and the evolving theory.

### 2.6 Interpretation of Data

During the interpretation phase, we closely examined how procrastination was justified by participants and how it influenced their academic and personal lives. These interpretations were informed by the participants' narratives, highlighting how procrastination functioned as both a maladaptive and sometimes adaptive strategy. The use of triangulation—cross‐referencing data from interviews, questionnaires, and academic records—helped enhance the credibility and depth of the emerging theory.

By following the systematic steps of Grounded Theory—open, axial, and selective coding—this study generated a data‐driven framework explaining the procrastinatory behaviors among students, which can serve as the foundation for further interventions.

### 2.7 Materials

#### 2.7.1 Tuckman Procrastination Scale

This scale was designed, developed, and standardized by David Tuckman at the University of Toronto in 2001 to assess students' procrastination. It consists of 16 items and is scored objectively. Furthermore, Mostafavi (2010) reported a reliability coefficient of 0.86 in her dissertation. This instrument utilizes a 5‐point Likert scale, where higher scores indicate increased levels of procrastination. Scores higher than 48 indicate high levels of procrastination.

### 2.8 Evolution of Researchers' Standpoint

As the research progressed, the initial focus on identifying procrastination as a maladaptive behavior expanded to a more nuanced understanding. Through the qualitative phase, we discovered that procrastination can sometimes serve as an adaptive coping mechanism in certain contexts (e.g., managing emotional distress or academic pressure). This insight influenced the development of the intervention package, which incorporated elements addressing both emotional regulation and motivational enhancement. Drawing on Sharlene Hesse‐Biber's work on mixed methods designs, we recognized that combining qualitative insights with quantitative validation allowed us to better understand not only the existence of procrastination but also its underlying causes and potential solutions. This iterative process of theory building followed by intervention testing highlights the strength of the mixed‐methods approach, aligning with the journal's focus on qualitative research.

### 2.9 Summary of the Content of the Designed Training Sessions

Summary of the content of the designed training sessions was as follows: In Session 1, participants are introduced to each other, the facilitator, and the concept of procrastination, including its various domains. They identify tasks they tend to procrastinate on and the pleasurable behaviors they use to alleviate the discomfort of uncompleted tasks. They are also encouraged to explore and articulate the justifications they use to maintain a positive self‐image despite procrastination (Cognitive‐behavioral approach). In Session 2, ineffective rules and beliefs underlying procrastination are explained, and participants identify the irrational beliefs specific to their own experiences. Both positive and negative consequences of procrastination are explored (Cognitive‐behavioral approach). In Session 3, participants explore the procrastination cycle and visualize their unique patterns, while identifying strategies to break these cycles. Cognitive distortions that contribute to procrastination are also challenged (Cognitive‐behavioral approach). In Sessions 4 and 5, mindfulness techniques like mindful breathing and body scans are used to reduce internal causes of procrastination, such as anxiety, depression, and fatigue (Mindfulness approach). In Session 6, assertiveness training and self‐efficacy enhancement help participants manage negative thoughts and identify their personal talents and abilities. Goal‐setting techniques are introduced to promote motivation for procrastinators (Motivational intervention). In Sessions 7 and 8, mediating and fundamental beliefs related to family dynamics, parenting characteristics, and personal traits that contribute to procrastination are addressed (Cognitive‐behavioral approach). In Session 9, factors that perpetuate procrastination, such as virtual distractions, fatigue, and lifestyle issues (including the impact of the pandemic), are discussed (Cognitive‐behavioral approach). In Sessions 10 and 11, participants engage in behavioral activation, learn effective coping strategies, practice time management and planning, and gain mindfulness of fleeting emotions (Cognitive‐behavioral and mindfulness approach). In Session 12, a summary of the program is provided, and questions are answered.

### 2.10 Procedure

All ethical considerations, including informed consent, the right to withdraw from the study, and the maintenance of anonymity and confidentiality throughout the study, were adhered to. For the quantitative portion, a quasi‐experimental pre‐test‐post‐test control group design was employed. A multi‐stage random sampling technique was used. Initially, faculties were randomly selected. Subsequently, the Tuckman (2001) was randomly distributed to 1000 students from Persian Gulf University, stratified by faculty, major, and year of study. From this pool, 70 individuals who were identified as procrastinators and met the required criteria were selected and randomly assigned to either the experimental group (*n* = 35) or the control group (*n* = 35).

Participants assigned to the control group did not receive any structured procrastination‐focused training during the study period. They continued their usual academic routine (treatment as usual within the university setting) and only participated in the assessment sessions (pre‐test and post‐test). To minimize contamination, control participants were asked not to attend other procrastination‐reduction workshops or psychological training programs during the intervention period. After completion of the post‐test assessment, the control group was offered the opportunity to receive the same training package. Participants in the experimental group received the grounded‐theory‐based procrastination treatment package delivered in 12 sessions, as summarized in the “Summary of the content of the designed training sessions” section. A one‐way analysis of covariance (ANCOVA) was conducted using SPSS version 26 to analyze the data.

## 3 Study 1: Qualitative Research

### 3.1 Results

This study involved semi‐structured interviews with 22 undergraduate participants. The research findings focused on attitudes toward procrastination, the underlying causes, and contributing factors, strategies employed to cope with procrastination, and the associated consequences. Line‐by‐line coding was performed after transcribing the interviews. Each statement was assigned a code based on the concepts it covered. The codes were created based on the participants' own words or the researcher's interpretation of the concepts underlying the data. The codes were compared, and an initial categorization of codes was performed by grouping similar codes. Key concepts and categories that explain various aspects of the participants' experiences were identified. At this point, 1159 codes have been formed. During the analysis, the categories and the data were constantly compared and refined. In the open coding stage, 147 concepts, 28 subcategories, and 11 categories were developed. Eventually, through selective coding, the core category of the research was identified.

**FIGURE 1:**
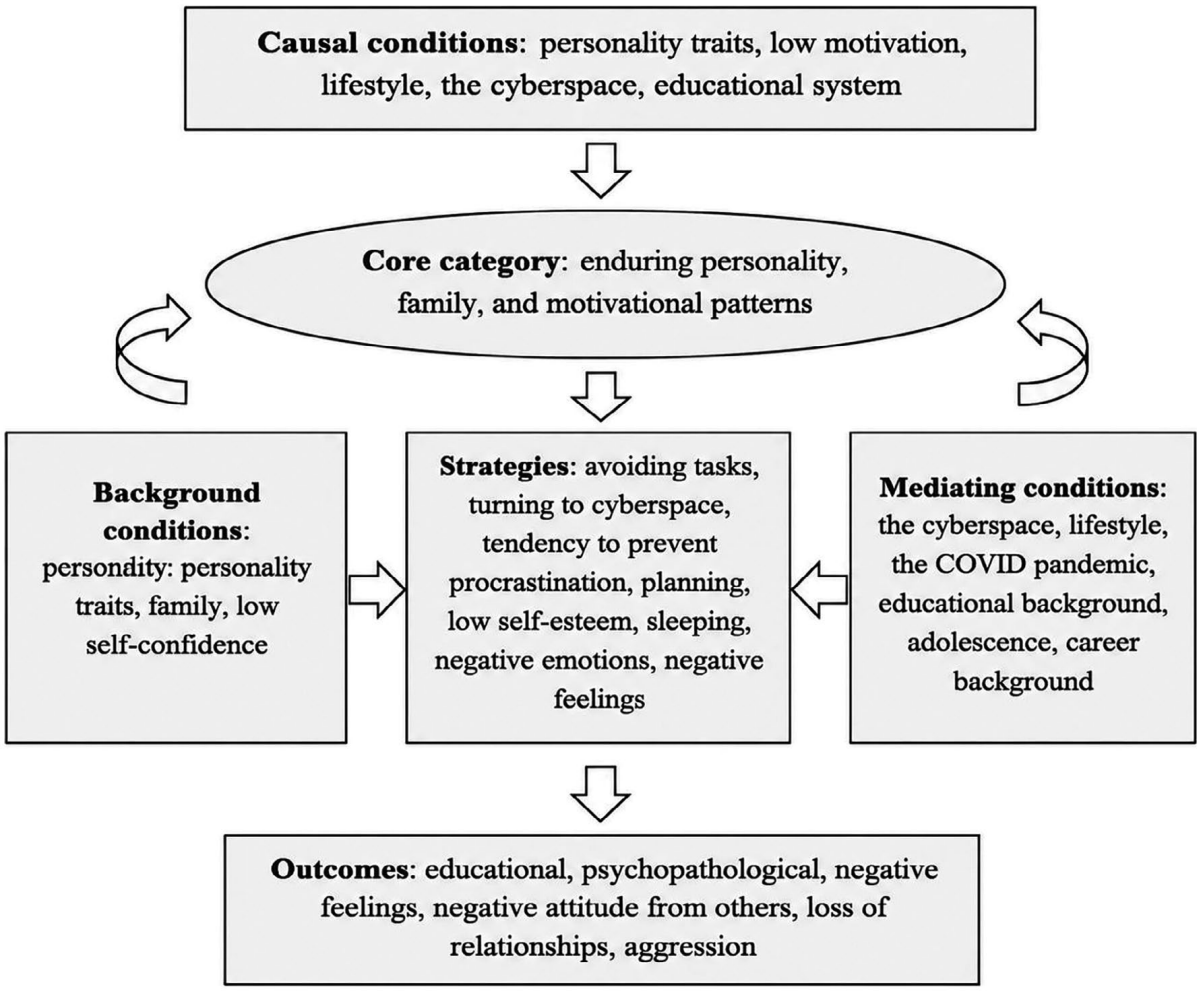
Paradigmatic model of procrastination development. Integrates the qualitative categories into a paradigmatic grounded model. The model links causal conditions (e.g., personality traits, low motivation, lifestyle, cyberspace, educational system) with background and mediating conditions (e.g., family dynamics, low self‐confidence, COVID‐related changes, educational background), which shape the selection of strategies (e.g., avoidance, cyberspace use, planning, emotion‐oriented responses) and lead to multiple outcomes (educational, psychopathological, negative feelings, interpersonal costs). At the center of this process, the core category—enduring personality, family, and motivational patterns—captures the stable configurations that maintain procrastination over time.

To view the Categories, subcategories, and concepts regarding the causes of procrastination, refer to Table 1, the factors of procrastination to Table 2, the coping mechanisms of procrastination to Table 3, the outcomes of procrastination to Table 4, and the Paradigmatic model of procrastination development to Figure 1.

**TABLE 1:** Categories, subcategories, and concepts regarding the causes of procrastination.

Sub‐concepts	Basic concepts	Main categories
Internal factors	Personality traits	Low self‐confidence, anxiety, low resilience, stubbornness, depression, irritability, inability to say no, perfectionism, fear of failure, fear of being viewed negatively, avoiding novel situations, romantic relationships, and irresponsibility
	Lack of motivation	Disappointment, lack of motivation, and disinterest
	Lifestyle	Oversleeping, irregular sleep patterns, feeling unwell due to lack of sleep, insufficient sleep the night before, hobbies, and social gatherings
External factors	The cyberspace	Overuse of cyberspace, late‐night hobbies, and mobile phone usage
	Educational system	Disinterest in one's field of study, boring classes, educational quality, and dormitory life

**TABLE 2:** Categories, subcategories, and concepts regarding the factors of procrastination.

Sub‐concepts	Basic concepts	Main categories
Background factors	Personality traits	Perfectionism, fear of failure, variety‐seeking, low resilience, obsession, anxiety, laziness, disorganization, depression, fear of judgment, habituation, irresponsibility, impracticality, stubbornness, forgetfulness, passivity, illusion of talent, staying in the safe zone, and ignoring family advice
	Family	History of procrastination, family influence, lack of emotional support, lack of verbal support, lack of supervision, and parents' procrastination
	Low self‐confidence	Decision‐making, sense of inefficacy, and uncertainty
Prolonging factors	The cyberspace	High Internet use, mobile phone usage, watching movies, Internet usage, and dependence on cyberspace
	Lifestyle	Craving sleep, lack of sleep the night before, going out with friends, attending gatherings, playing instead of working, prioritizing sleep over work, irregular sleep patterns, disturbed sleep, avoiding challenges, and lack of family education
	The COVID pandemic	Staying home, increased procrastination, and online classes
Revealing factors	Education	Being late to classes, delaying studying, repeated absence, lack of interest in one's field of study, increased procrastination due to lack of force, and academic pressure
	Adolescence	Higher relative procrastination in adolescence, increased procrastination with age, and the impact of puberty
	Career	Ignoring career opportunities, and irresponsibility

**TABLE 3:** Categories, subcategories, and concepts regarding the coping mechanisms of procrastination.

Sub‐concepts	Basic concepts	Main categories
Avoidance strategies	Avoiding tasks	Making excuses, doing unrelated things, evading tasks, and postponing tasks
	Turning to cyberspace	Using mobile phones, watching movies, and overusing cyberspace
Confrontation strategies	Tendency to avoid procrastination	Tendency to finish tasks, thinking about working, motivating oneself to work, and mental preparation for working
	Planning	Notes, organizing, and planning
Surrender strategies	Low self‐esteem	Feeling inefficacious, feeling unhelpful, feeling defeated, feeling incapable, feeling futile, low self‐confidence, and comparison with others
	Sleeping	Preferring sleep to completing tasks, and resting
Emotion‐oriented strategies	Negative emotions	Self‐hatred and self‐dissatisfaction, fear, aggression, anger, self‐criticism, guilt, sadness, and shame
	Negative feelings	Guilt, stress, anxiety, depression, restlessness, and regret

**TABLE 4:** Categories, subcategories, and concepts regarding the outcomes of procrastination.

Sub‐concepts	Basic concepts	Main categories
Personal outcomes	Educational	Academic decline, repeated absence, dropping courses, academic failure, and prolonging studies
	Psychopathological	Stress, anxiety, and depression
	Negative feelings	Regret, remorse, sadness, self‐criticism, self‐hate, disappointment, guilt, and hatred
Interpersonal outcomes	Negative attitude from others	Ridicule from friends, negative perceptions, and inefficacy
	Loss of relationships	Losing friends and having fewer relationships
	Aggression	Aggression even in the face of reasonable behavior, and irritability towards others

As shown in Table 1, students described procrastination as arising from both internal and external causal conditions. Internal causes primarily involved personality‐related vulnerabilities (e.g., low self‐confidence, anxiety, perfectionism, fear of failure) as well as motivational and lifestyle‐related disruptions. External causes emphasized contextual triggers such as cyberspace‐related distractions and features of the educational system (e.g., disinterest in the field of study, boring classes). Together, these conditions form the initial “causal layer” of the grounded model.

Table 2 extends the causal layer by identifying broader background conditions that shape students' vulnerability to procrastination over time. In addition to individual characteristics (e.g., fear of failure, low resilience, disorganization), participants emphasized the role of family climate (e.g., limited emotional/verbal support, low supervision, and family history of procrastination). These background factors help explain why procrastination may persist even when students recognize its negative consequences.

As presented in Table 3, participants reported multiple strategy patterns in response to procrastination. Avoidance‐oriented strategies involved delaying tasks and turning to cyberspace, whereas confrontation‐oriented strategies included increasing readiness to act and using planning behaviors. In addition, some strategies reflected surrender and emotion‐oriented responses (e.g., low self‐esteem, sleeping, negative emotions and negative feelings), suggesting that coping is not purely behavioral but also strongly emotional and self‐evaluative.

Table 4 summarizes the consequences reported by students across personal and interpersonal domains. Educational consequences (e.g., academic decline, repeated absence, dropping courses) were frequently accompanied by psychopathological distress (stress, anxiety, depression) and self‐directed negative affect (regret, guilt, self‐criticism). Interpersonally, students described deterioration in others' perceptions, relationship losses, and irritability/aggression, indicating that procrastination functions as a cycle with reinforcing costs across multiple life domains.

### 3.2 Discussion

Previous studies on procrastination have generally focused on its etiology and ways to counter it (Kurniawan 2024; Fridayani 2023; Yastibas 2020). This research, diverging from previous research, utilizes the grounded theory methodology to study how procrastination develops. In this study, procrastination is viewed as a complex and dynamic system, considering cultural, social, organizational, and individual influences. This study also identifies the various factors that contribute to procrastination, examines their interactions, and explores their impact on procrastination. With this novel approach, the current study has achieved a comprehensive understanding of the process of procrastination and provides strategies to prevent it, drawing from this profound comprehension. Therefore, by offering a new perspective, this study can help in developing more effective strategies to combat procrastination.

Causal factors are events that influence phenomena (Strauss and Corbin 1998). As for the first question of this research regarding the factors that cause procrastination, five subcategories were identified: personality traits, lack of motivation, lifestyle, cyberspace, and the educational system.

Procrastination is a personality trait in which anxiety plays a prominent role. The ability to concentrate, make choices, and feel motivated is reduced when someone is worried. As such, an individual's lack of motivation can be attributed to anxiety, which leads to procrastination, as pointed out by Kranjec et al. (2016) in their work. Therefore, procrastination serves as a means of escaping from stress. In simple terms, an individual who procrastinates is simply trying to avoid the unpleasant feeling of anxiety.

Lack of motivation is a primary concern among self‐related factors, possibly triggered by various issues like anxiety, depression, or increased stress. When an individual lacks the necessary motivation to fulfill their obligations and tasks, they may encounter obstacles in their completion, leading to procrastination. Regarding the correlation between personal perception of assignments and procrastination, it is posited that effective internal motivation and profound comprehension occurring simultaneously during the acquisition of a new topic result in comprehensive understanding and mastery of the subject matter. Consequently, a surge in interest, enjoyment, satisfaction, and eagerness toward learning topics ensues, thereby bolstering enthusiasm. Conversely, engaging with subjects in a perfunctory manner, devoid of interest, tends to amplify tendencies toward procrastination (Klassen et al. 2008).

Insufficient sleep the night before is the primary factor among those related to lifestyle. Sleep deprivation may lead to procrastination, as individuals who suffer from insufficient sleep may experience fatigue, diminished concentration, and reduced motivation. Therefore, sufficient and timely sleep plays a crucial role in maintaining motivation and efficiency. The overuse of cyberspace, late‐night hobbies, and mobile phone usage may lead to procrastination. Among these factors, significant emphasis is placed on the overuse of cyberspace. From a cognitive perspective, procrastination is not driven by a lack of drive or initiative but rather by the allure of alternative tasks that capture the attention of the individual in question. In academic contexts, procrastination specifically indicates that the motivation to engage in reading tasks is overshadowed by other competing thoughts or activities, ultimately affecting one's academic performance (Lian et al. 2018). Through this explanation, it is evident that involving individuals in cyberspace can lead to increased incentives and faster gratifications. This includes establishing friendships, seeking validation from peers (through “likes”), following friends, staying updated with the news, and sharing visually appealing images and videos.

The primary concern within the educational system's framework related to external factors is the lack of interest in the field of study. The configuration and implementation of educational systems play a crucial role in fostering procrastination tendencies. A lack of interest in the subject matter can lead to apathy toward educational issues, including academic procrastination. A student who lacks interest in their academic discipline may find themselves devoid of the necessary drive to engage with their studies effectively, potentially resulting in structural deficiencies within their field of study and subsequent academic anxiety. It follows logically that without the motivation to delve into subject matters pertaining to the field, a meaningful and productive engagement within the classroom setting cannot be achieved, leading to disengagement and academic procrastination (Sneyers and de Witte 2017).

The second research question pertained to background factors, which are factors that accumulate at a specific time and place to shape a set of conditions to which a person reacts (Strauss and Corbin 1998). Five subcategories of background factors were identified: personality traits, family, parenting styles, low self‐confidence, and lack of motivation.

Personality traits can significantly contribute to the phenomenon of procrastination, which is characterized by a recurrent pattern of delaying tasks. Procrastination is a complex behavior influenced by various factors, including an individual's disposition, family dynamics, self‐confidence, self‐efficacy, as well as feelings of despair and perfectionism. Personality traits play a significant role in fostering procrastination. For instance, the trait of perfectionism is closely linked to procrastination because individuals who exhibit perfectionistic tendencies often set exceedingly high standards for themselves. These high expectations lead to increased stress levels, as individuals feel pressured to complete tasks perfectly. Consequently, individuals may resort to avoidance or neglecting responsibilities as a means of alleviating this pressure. Moreover, perfectionists consistently experience discontent with their own performance and preferred course of action, choosing to abandon tasks rather than execute them inadequately. This particular phenomenon remains a pivotal factor contributing to procrastination tendencies among individuals (Kurtovic et al. 2019).

The family can significantly impact the development of procrastination habits. When a history of procrastination exists within the family, individuals are more inclined to exhibit similar behavioral patterns. This observation can be attributed to the family serving as a fundamental social institution, where the dynamics among its members hold substantial significance. The quality of relationships within the family unit establishes an atmosphere that influences the emergence of thoughts, emotions, and behaviors, thereby interacting with the established family dynamics. Consequently, parental procrastination behaviors can exert a reciprocal influence on the procrastination tendencies displayed by their children (Gündüz 2020).

Among the factors contributing to low self‐confidence, the absence of a sense of self‐efficacy is a primary concern. This observation suggests that individuals with low self‐efficacy in academic matters tend to lean toward surrender and avoidance. Perpetually doubting their abilities, they constantly worry about not meeting expectations and try to justify possible failures by embracing a sense of ineffectiveness (Steel and Klingsieck 2016).

The intervening factors within this study were identified as eight concepts: perpetuating factors encompassing cyberspace, burnout, lifestyle, and the COVID pandemic, and revealing factors including academic background, interpersonal communication, adolescence, and career background.

A predominant factor in perpetuating procrastination is the excessive use of cyberspace. This overreliance can consequently contribute to procrastination behaviors. Individuals who heavily rely on cyberspace often face challenges in managing their time. By dedicating most of their time to virtual platforms, individuals find themselves ill‐prepared to participate in academic pursuits, professional duties, physical activities, and even relaxation. Consequently, as students increasingly immerse themselves in virtual realms, they experience a loss of control over their internet and cyber usage. This lack of restraint results in an inability to stop or control their reliance on virtual spaces (Hinsch and Sheldon 2013).

The primary lifestyle factor contributing to procrastination is oversleeping. Lifestyle factors play a crucial role in influencing procrastination behaviors. This observation can be attributed to the current increase in the number of social networking applications available on various technological devices, which results in people spending extended periods on these platforms. Consequently, individuals, particularly students, who are engrossed in social networking activities often experience disruptions in their sleep patterns. Some individuals may prefer daytime sleeping because they do not get enough rest at night, which leads to not having enough time to fulfill their responsibilities, thus encouraging procrastination tendencies (Levenson et al. 2016).

A significant concern in the current academic environment is how procrastination has been exacerbated by the COVID‐19 pandemic. Given the novel challenges posed by this pandemic, it is urgent to address its influence on perpetuating procrastination. There are a variety of daily obligations and duties that individuals have to fulfill, each being time‐bound and deadline‐oriented. This means that one can postpone their assignments despite having planned their time, energy, and other resources meticulously due to stress and anxiety. Consequently, the challenging environment created by the epidemic hindered students from completing their academic tasks, compelling them to procrastinate (Steel 2007).

The revealing factors include one's educational background, interpersonal relationships, adolescence, and career background. Notably, a primary concern within the realm of education pertains to repeated absences in academic settings as a prominent indicator of procrastination. A plausible interpretation of this observation suggests that students' commitment to their academic pursuits, along with their ability to navigate common academic challenges, plays pivotal roles in fostering consistent classroom attendance and overall school engagement. In addition, students' academic commitment is a potential predictor of absenteeism in classrooms and universities. Those with less dedication to their studies tend to skip classes, which then leads to a cycle of procrastination. In contrast, individuals who are more focused on their education are less likely to fall into this trap. This reveals how academic integrity affects students' tendency to procrastinate in school (Martin and Marsh 2008).

High levels of procrastination during adolescence are the primary adolescence‐related factor in revealing procrastination. The adolescent is going through a transition from childhood into adulthood and puberty. The society demands them to find their place as a grown, independent, and creative individual, change their relationships with their peers and adults, develop sexual and career adaptations, and moreover, to try and create their own identity. The stresses of this stage of life decrease the adolescent's motivation. One of the consequences of this decreased motivation is procrastination (Steel 2007).

Failure to execute designated tasks is a prominent manifestation of procrastination in professional settings, serving as a key indicator of this behavioral tendency. Individuals inclined toward procrastination are often deterred from undertaking their assigned responsibilities due to a lack of motivation or interest in the tasks. Consequently, they may perceive procrastination and non‐compliance as the sole viable course of action. Alternatively, individuals exhibiting perfectionist traits within the workplace may withhold their participation in assigned tasks, deeming them incompatible with their professional standing.

The third research question addresses strategies that are based on actions and reactions to control and manage the phenomenon under study. Strategies are goal‐oriented and occur for a reason (Strauss and Corbin 1998). Ten concepts related to strategies were identified: avoiding tasks, self‐persuasion, turning to cyberspace, tendency to avoid procrastination, planning, low self‐esteem, sleeping, negative emotions, negative feelings, and temporary positive feelings.

The main tactic is to find justifications for not doing the work, so it is an integral part of the strategies of avoidance. Procrastination develops internal consequences that include frustration, regret, self‐accusation, guilt, and moral doubts. A person subconsciously knows that postponing the execution of current activity in no way leads to something good; that is why they feel guilt and moral frustration. Thus, the ones who are inclined to procrastination mostly find justifications for the failure to complete the given assignments in order to overcome the negative emotions and to attend to their duties (Oflazian and Borders 2023).

The use of a mobile phone is a primary source of procrastination in cyberspace and a key element of avoidance strategies. According to the behavioral theory, procrastination is based on Skinner's principles of reinforcement and punishment. According to this theory and model, tasks that are not followed by immediate reinforcement are likely to be delayed or avoided by the person. When comparing the two activities of using social networks and attending to academic tasks, we observe that individuals engaged in social networks receive interesting content such as texts, images, videos, news updates, and friendship requests. These elements serve as reinforcement and motivation for individuals to invest more time on the platform. Conversely, the person engaging with their academic tasks does not receive immediate reinforcement, or if they do, it is less significant than those offered on social networks. Therefore, individuals may spend more time on social networks and using their phones, leading to delays in their academic tasks (Kaliba and Ambrožová 2021).

The tendency to fulfill duties can be considered the top priority in coping with procrastination. This strategy can have a positive impact on individuals' behavior and help reduce procrastination. Considering students' insight that procrastination has a negative impact on the level of learning and the current and future success of learners, delays in starting and completing tasks will lead to missing learning opportunities. This can result in individuals pursuing studying and task completion at inappropriate times, always under time constraints. Such behavior disrupts the learning process, causing a decrease in accuracy, an increase in stress, and numerous mistakes in task completion and the learning process. Therefore, awareness of these negative consequences and the realization that procrastination may provide short‐term pleasure but lead to long‐term undesirable effects can motivate individuals to counteract this tendency by seeking solutions for task completion (Blikra 2022).

Planning also can be considered the top priority in coping with procrastination. By creating an effective and goal‐oriented plan tailored to specific objectives and tasks, individuals can stay informed about important dates and key tasks, thus optimizing their time and energy. This action can prevent delays and procrastination in task completion and help individuals to act in an organized and effective manner. It seems that having a specific goal is a crucial individual factor in academic progress or decline. It is evident that students who set appropriate goals for their studies will experience more success than those who do not have a specific and suitable goal. Having the right goal prompts the student to plan how to achieve it and move in a predetermined direction (Watson 2001).

Low self‐confidence is a key surrender strategy associated with procrastination. Procrastination is a disabling factor that appears to have a strong relationship with anxiety and low self‐confidence. The more students neglect their responsibilities, the more they experience higher levels of anxiety, reduced self‐confidence, and a decreased willingness to engage in their academic and social commitments. This can result in denial and passivity (Walters 2003). Maslow places self‐respect or self‐esteem at the third level of the hierarchy of needs, which includes dignity, integrity, progress, ability, competence, confidence, independence, and freedom. When these needs are met, the individual feels valuable, capable, productive, and confident. If these needs are not met, the individual may feel inferior, helpless, weak, cold, and hopeless. This sense of hopelessness and coldness can lead to withdrawal from many daily activities and a decrease in self‐confidence, a phenomenon known as procrastination (Yang et al. 2021).

Preferring sleep over completing tasks is the primary surrender strategy among those related to sleep. Students with mastery goals welcome tasks, focus on developing skills, and strive to achieve their goals. Few external obstacles impede their progress. Therefore, they do not prefer sleep over completing their tasks unless there is a physical need for it. In other words, they procrastinate less. Students with performance‐avoidance goals strive to avoid negative consequences. When the consequences can be evaded, or in contrast, when individuals experience learned helplessness, they tend to procrastinate and stop their activities, or they turn to sleeping instead of completing their tasks (Howell and Watson 2007).

Self‐hatred and self‐dissatisfaction take center stage in the negative emotions subcategory of emotion‐oriented coping strategies. Academic procrastination is the most common form of procrastination among students, and it is viewed as a bad habit that many students struggle with in their academic endeavors. Procrastination in preparing for exams, finishing homework, and other important tasks can lead to negative mental outcomes, such as low self‐esteem, anxiety, guilt, and depression, as well as adverse educational outcomes like low grades and missing deadlines. These outcomes and the mental pressure that accompany them cause students to hate and be dissatisfied with themselves (Stoliker and Lafreniere 2015).

Anxiety is the main concept within the negative feelings subcategory of emotion‐oriented strategies. It can be said that individuals who perceive their tasks as threatening and anxiety—inducing will delay them. Procrastinators may experience lower levels of anxiety at the beginning of the semester by dedicating less time to studying. However, as deadlines draw near, their anxiety levels will increase. In other words, this anxiety resembles neuroticism. Therefore, anxious students may procrastinate due to the tension and aversion caused by the tasks (Yerdelen et al. 2016).

The fourth research question was regarding the outcomes. Eight concepts were identified: educational, psychopathology, negative feelings, lack of self‐confidence, negative attitude from others, loss of relationships, and aggression.

Academic decline is the primary concept within the educational subcategory of procrastination outcomes. Academic procrastination manifests in behaviors such as delaying homework or studying for an exam. A common example is delaying exam preparation until the night before, which leads to anxiety and rushing. The negative cognitive and emotional consequences of this make it a maladaptive behavior that can severely impact students' academic aptitude. Procrastinators live solely in the present, as if there is no tomorrow; they consider the future only an extension of the present moment. This sort of thinking can eventually disrupt their time management skills. Inefficient time management, low self‐confidence, concentration problems, low responsibility, anxiety, and fear of failure are all associated with the postponement of tasks. Since academic activities occur over a long and predetermined timespan, and academic success requires constant effort for at least a semester, ignoring academic tasks and prioritizing other activities can cause a decline in academic performance (Wusik and Axsom 2016).

Stress is the primary psychopathological outcome of procrastination. Students with motivational beliefs utilize adaptation patterns such as perseverance, positive affect, and problem‐solving strategies. Conversely, students who procrastinate experience a fear of failure, reduced perseverance, negative affect, and reduced use of problem‐solving strategies, all of which negatively impact their academic performance. If individuals explore their attitudes toward their fears of failure, success, being controlled by others, intimacy, separation, and so on, and identify what scares them, they are likely to begin their tasks with increased self‐confidence and stop procrastinating. Therefore, having high motivation and low stress, which result from using effective cognitive strategies, can reduce procrastination in students and consequently alleviate their stress. Otherwise, the procrastinator is likely to experience stress (Wang et al. 2010).

Regret is the primary concept within the subcategory of negative feelings among the personal outcomes of procrastination. Procrastinators admit to their wrongdoings and are always bothered by their bad habits. They struggle with feeling worthless and unpleasant. In other words, despite the temporary positive feeling induced by delaying their tasks, procrastinators will eventually start contemplating their actions. Through an analysis of their performance, they begin to feel regret (Ferrari 2001).

Negative perception by others is the primary interpersonal outcome of procrastination. Procrastination can lead others to form a negative perception of those who exhibit this behavior. Delaying tasks, failing to achieve goals, and neglecting commitments can lead others to develop a negative attitude toward the procrastinator. This can damage trust and relationships (Abramowski 2018).

Loss of relationships is another interpersonal outcome of procrastination. Procrastination can limit individuals' relationships. When students procrastinate and fail to complete their tasks, others may become disappointed in them and even end their relationships with them. This can cause tension, unease, and isolation between individuals, eventually reducing positive and healthy interactions (Klassen et al. 2010).

Irritability toward others is the primary concept in the aggression subcategory of personal outcomes of procrastination. Procrastination can cause irritability toward others because delaying tasks and neglecting responsibilities can lead to interpersonal conflict and create tension in relationships. This can then lead to aggression and impatience, eventually disrupting communication with others. Howell and Watson (2007) view procrastination as a self‐regulation disability that individuals employ when confronting life's challenges. Some view procrastination as the antithesis of motivation. With prolonged and repeated procrastination, individuals on one hand experience obsessions, and on the other hand feel disappointed and rejected due to malfunctioning in their tasks, leading to increased irritability.

Answering this study's overarching question, the core category comprises enduring personality traits, family dynamics, and motivational patterns. Such stable patterns and traits can influence the likelihood of procrastination. For example, individuals with traits such as indifference, dissatisfaction, or non‐compliance with organizational regulations are more likely to procrastinate. Family patterns, such as inadequate support for children or unstable marriages, can also increase the risk of procrastination. Individuals with low or unstable motivation are more likely to procrastinate. Therefore, analyzing and studying these patterns and traits can help shape a better understanding of procrastination and develop preventative measures.

### 3.3 Intervention Development and Qualitative‐To‐Quantitative Integration

The intervention package used in Study 2 was explicitly derived from the grounded theory findings generated in Study 1. Following open, axial, and selective coding, the qualitative results were summarized into core categories representing (a) causal and background conditions (e.g., personality‐related vulnerabilities, low motivation, lifestyle patterns, cyberspace distraction, family dynamics, educational context), (b) action/interaction strategies (e.g., avoidance, turning to cyberspace, planning, emotion‐oriented coping), and (c) consequences (educational, psychological, and interpersonal outcomes), organized within the paradigmatic model (Tables 1, 2, 3, 4; Figure 1).

To translate these qualitative themes into a structured intervention, we used a systematic mapping procedure. First, each major grounded‐theory theme was converted into a change target (e.g., maladaptive beliefs, avoidance cycles, emotion regulation difficulties, digital distraction, motivational fragility, family‐related core beliefs, lifestyle dysregulation). Second, for each target, we selected corresponding intervention strategies (psychoeducation, cognitive restructuring, mindfulness skills, assertiveness/self‐efficacy enhancement, motivational goal‐setting, behavioral activation, time‐management/planning, and relapse prevention). Third, we transformed strategies into concrete session components (in‐session exercises, group discussions, and between‐session homework tasks). Finally, the resulting session plan was reviewed and refined by experts in psychology to ensure clarity, feasibility, and content validity. The final mapping of qualitative themes to intervention components is provided in Table 5, and the session‐by‐session content is summarized below.

**TABLE 5:** Mapping grounded theory themes (Study 1) to intervention components (Study 2).

Grounded‐theory theme/category (Study 1)	Evidence in Study 1 (tables/figure)	Design implication (what must change?)	Intervention component(s) in Study 2	Example in‐session activities	Example homework tasks
Personality‐related vulnerabilities (e.g., low self‐confidence, anxiety, perfectionism, fear of failure)	Internal factors/personality traits	Reduce catastrophic thinking, fear‐based avoidance; strengthen self‐efficacy	CBT cognitive restructuring (Sessions 2–3), mindfulness for distress tolerance (Sessions 4–5), assertiveness/self‐efficacy (Session 6)	Thought record; identifying cognitive distortions; mindful breathing/body scan	Daily thought log; 10–15 min mindfulness practice; graded task engagement
Low motivation/disinterest	Lack of motivation	Increase intrinsic motivation; clarify values; strengthen goal commitment	Motivational intervention/goal‐setting (Session 6)	Values clarification; SMART goals; “why this matters” discussion	Weekly goals worksheet; commitment contract; progress monitoring
Lifestyle dysregulation (sleep, fatigue, irregular routines, social gatherings)	Lifestyle factors	Stabilize routine; reduce fatigue‐driven delay; increase activation	Lifestyle & fatigue management (Session 9), behavioral activation & planning (Sessions 10–11)	Activity scheduling; sleep hygiene micro‐lesson; energy management plan	Sleep/wake tracking; activity schedule; “2‐min start” practice
Cyberspace distraction/mobile phone	External factors: cyberspace and prolonging factors	Reduce digital‐triggered avoidance; increase stimulus control	Managing virtual distractions (Session 9), time management & coping (Sessions 10–11)	Trigger mapping (apps/contexts); digital boundaries; “if–then” plans	Phone‐use diary; app limits; focus blocks (Pomodoro)
Educational‐system triggers (boring classes, low quality, dormitory life)	Educational system	Improve study tactics; reduce learned helplessness; increase proactive behaviors	Psychoeducation & cycle analysis (Sessions 1–3), assertiveness/problem‐solving (Session 6), planning/time management (Sessions 10–11)	Procrastination cycle mapping; problem‐solving steps; study planning	Weekly plan; task breakdown; help‐seeking script
Family dynamics & foundational beliefs	Table 2: family factors; background conditions list	Reframe core beliefs rooted in family patterns; reduce self‐criticism	Fundamental/mediating beliefs & family dynamics (Sessions 7–8)	Core belief identification; schema‐linked discussion; compassionate reappraisal	Core‐belief worksheet; self‐compassion letter; communication practice
Avoidance strategies (postponing, excuses, doing unrelated things)	Table 3: avoiding tasks; session 1 “justifications”	Break avoidance cycle; replace excuses with action cues	Psychoeducation + identifying justifications (Session 1), cycle visualization (Session 3), behavioral activation (Sessions 10–11)	“Excuse audit”; action triggers; exposure‐to‐start	“Start‐before‐ready” task; avoidance log; graded exposure plan
Emotion‐oriented coping (guilt, stress, anxiety, regret, shame)	Table 3 negative emotions/feelings; outcomes psychopathological/negative feelings	Improve emotion regulation; tolerate discomfort without escaping	Mindfulness skills (Sessions 4–5; 10–11)	Body scan; urge surfing; observing emotions	Daily mindfulness; emotion diary; “name it to tame it”
Planning as protective coping	Table 3 planning	Build consistent planning and implementation habits	Time management & planning (Sessions 10–11)	Task breakdown; weekly calendar; prioritization matrix	Weekly planning sheet; implementation intentions; review & adjust
Core category: enduring personality, family, and motivational patterns	Core category explicitly stated	Consolidate gains; relapse prevention around enduring patterns	Integration & relapse prevention (Session 12)	Personal model recap; warning signs; maintenance plan	Maintenance checklist; “if relapse then…” plan; booster goals

## 4 Study 2: Quantitative Research

Following the development of a procrastination intervention package for students based on grounded theory in the qualitative phase, the quantitative hypothesis regarding the impact of the developed package on students' procrastination was tested using a one‐way analysis of covariance (ANCOVA) in SPSS version 26.

Table 6 reports the pre‐test and post‐test descriptive statistics for the experimental and control groups. Because higher scores on the Tuckman Procrastination Scale indicate higher procrastination (and scores above 48 indicate high procrastination), a decrease in the mean score from pre‐test to post‐test is interpreted as improvement.

**TABLE 6:** Means and standard deviations of procrastination in the pre‐test and post‐test for the experimental and control groups.

Group	Test	Mean	SD
Experiment	Pre‐test	48.22	3.80
	Post‐test	37.77	4.89
Control	Pre‐test	48.42	3.54
	Post‐test	43.71	3.97

As shown in Table 6, the experimental and control groups' mean procrastination scores are reported at pre‐test and post‐test. The experimental group demonstrates a larger improvement from pre‐test to post‐test relative to the control group, consistent with the expected direction of change after receiving the intervention. These descriptive patterns are subsequently tested inferentially using ANCOVA (Table 7), which evaluates whether post‐test group differences remain significant after controlling for baseline (pre‐test) procrastination.

**TABLE 7:** Effect of the intervention on students' procrastination (one‐way analysis of covariance).

	The sum of squares (SS)	Degree of freedom	Mean square	*F*‐value	*p*
Intergroup	15.577	1	15.577	98.19	0
Intragroup	82.1963	68	88.28		
Total	98.2540	69			

The homogeneity of variance in procrastination scores between the experimental and control groups was examined using Levene's test. Given the non‐significant result of Levene's test (*p* = 0.34), it can be concluded that the variances of procrastination scores are homogeneous between the experimental and control groups. Therefore, since the assumption of homogeneity of variances, a prerequisite for conducting a one‐way ANCOVA, has been met, this analysis was performed.

A one‐way ANCOVA was conducted to compare post‐test procrastination between groups while statistically controlling for baseline (pre‐test) procrastination. As reported in Table 7, the group effect was significant (*F* = 19.98, *p* < 0.001), indicating that the intervention produced a statistically meaningful reduction in procrastination beyond what could be attributed to baseline differences. To improve interpretability, we also report the adjusted post‐test means for each group and an effect size (e.g., partial *η* ^2^) alongside the F statistic.

### 4.1 Discussion

Previous studies on procrastination have generally focused on its etiology and ways to counter it (Kurniawan 2024; Fridayani 2023; Yastibas 2020). Building on this literature, the present study diverges by using a mixed‐methods grounded theory approach to explain how academic procrastination develops as a dynamic process and to translate that explanation into an intervention package that can be empirically tested. This approach aligns with the broader view that procrastination is a prototypical self‐regulatory failure, strongly intertwined with motivational beliefs, self‐efficacy, and emotional distress (Steel 2007; van Eerde 2003). Importantly, our grounded model extends correlational findings by clarifying mechanisms—how personal vulnerabilities, contextual triggers, and coping strategies interact and reinforce one another over time.

The qualitative findings (Tables 1, 2, 3, 4; Figure 1) indicate that procrastination is not a single‐factor behavior but a multi‐layered system shaped by causal conditions, background and mediating conditions, and action/interaction strategies, leading to consequences across academic, psychological, and interpersonal domains. This configuration is consistent with process‐oriented accounts emphasizing that procrastination frequently functions as short‐term mood regulation under task aversiveness or academic pressure (Sirois 2023; Steel 2007). In other words, delaying may temporarily reduce negative affect, but the relief is short‐lived and tends to maintain the cycle, ultimately worsening stress and functioning.

Causal factors are events or conditions that influence a phenomenon (Strauss and Corbin 1998). In response to the first research question, our results identified causal conditions spanning internal and external domains, including personality‐related vulnerabilities, lack of motivation, lifestyle‐related disruption, cyberspace‐related distraction, and elements of the educational system. This is consistent with evidence that procrastination is linked to motivational and self‐regulatory difficulties, low self‐efficacy, and maladaptive beliefs (Ma et al. 2023; Steel 2007; van Eerde 2003). For instance, anxiety can diminish concentration and perceived control, which in turn undermines motivation and increases avoidance—an interpretation also reflected in prior work (Kranjec et al. 2016). Similarly, disinterest in the field of study and negative classroom experiences can reduce intrinsic engagement, facilitating procrastination through lowered academic value and increased academic pressure (Sneyers and de Witte 2017).

Background factors accumulate to create the conditions in which individuals respond to tasks and stressors (Strauss and Corbin 1998). Our findings highlight that procrastination is embedded in broader contexts, including family dynamics (e.g., low emotional support, low supervision, intergenerational patterns), personality‐related history, and low self‐confidence. Such factors plausibly shape persistent self‐regulatory vulnerabilities and may explain why procrastination becomes entrenched rather than episodic. In addition, mediating conditions—particularly cyberspace use, lifestyle pressures, and contextual shifts associated with the COVID pandemic—appear to intensify procrastination by increasing distraction, fatigue, and task aversiveness. This aligns with literature suggesting that digital environments can compete with academic tasks by offering rapid reinforcement and immediate gratification, thereby strengthening avoidance and delay (Kaliba and Ambrožová 2021; Lian et al. 2018).

The coping patterns identified in Table 3 suggest that procrastination is maintained through a combination of avoidance‐oriented and emotion‐oriented strategies (e.g., turning to cyberspace, sleeping, negative emotional cycles), alongside less frequent confrontation strategies such as planning and readiness to act. These findings support the view that procrastination is often a coping response to aversive internal states, rather than simply “poor time management” (Sirois 2023). Planning and goal‐directed organization emerged as protective strategies, consistent with frameworks emphasizing the importance of clear goals and structured self‐regulation for academic progress (Watson 2001). In contrast, avoidance and cyberspace engagement appear to provide short‐term relief while reinforcing the delay cycle, consistent with behavioral reinforcement explanations and modern accounts of distraction‐based procrastination (Kaliba and Ambrožová 2021; Steel 2007).

Table 4 demonstrates that procrastination produces multidimensional costs. Educational consequences (academic decline, repeated absence, dropping courses) were frequently accompanied by psychopathological distress (stress, anxiety, depression) and negative feelings (guilt, regret, self‐criticism). Prior research likewise indicates that procrastination is reliably associated with increased distress and impaired functioning (Sirois 2023; Steel 2007). In our findings, stress emerged as a primary psychopathological outcome, consistent with evidence that maladaptive motivational beliefs and reduced problem‐solving strategies may amplify academic strain among procrastinators (Wang et al. 2010). Regret was central among negative feelings, reflecting the common pattern in which temporary relief from delaying is later replaced by self‐blame and dissatisfaction (Ferrari 2001). Interpersonally, negative perceptions by others and relationship losses were prominent, suggesting that procrastination can erode trust and social functioning over time (Abramowski 2018; Klassen et al. 2010). Irritability and aggression also appeared as interpersonal outcomes, consistent with views linking procrastination to self‐regulation difficulties under stress (Howell and Watson 2007).

Answering the overarching question, the core category comprises enduring personality traits, family dynamics, and motivational patterns. This core captures the stable configurations that predispose individuals to procrastination and maintain it across contexts. Conceptually, this aligns with meta‐analytic evidence that procrastination is strongly tied to stable self‐regulatory vulnerabilities and belief systems (Steel 2007; van Eerde 2003). The grounded theory contribution here is not only listing correlates, but clarifying how these enduring patterns interact with mediating conditions (e.g., cyberspace distractions, lifestyle disruptions) to shape strategies and outcomes.

This study aimed to design, develop, and validate an educational package for treating procrastination in students, grounded in grounded theory. The intervention was derived from semi‐structured interviews and refined through expert validation before implementation. The results of the ANCOVA indicated that the intervention package significantly reduced academic procrastination among students. This finding is consistent with prior work showing that structured psychological interventions can reduce academic procrastination (Glick and Orsillo 2015; Wang et al. 2015). It also converges with broader evidence that CBT‐oriented approaches can yield meaningful improvements in procrastination‐related outcomes (Rozental et al. 2015).

A key interpretive strength of the present study is that the intervention was designed to match the mechanisms identified in the qualitative model. The package targeted cognitive processes (e.g., irrational beliefs and self‐justifications), emotional regulation skills (e.g., mindfulness‐based practices), and motivational/goal‐setting components. In line with theory and prior findings, procrastinating students often exhibit fear of failure, low self‐esteem and self‐efficacy, and a pessimistic outlook on the future; their cognition may be dominated by negative thoughts that undermine motivation and task engagement (Browne 2016; Steel 2007). By cultivating non‐judgmental awareness of thoughts and emotions, the intervention plausibly improved emotion regulation and reduced academic stress, thereby supporting task initiation and follow‐through. Moreover, through cognitive restructuring and relaxation‐related techniques, the intervention may have enhanced attention and concentration during study, which is particularly relevant given that concentration difficulties are frequently implicated in academic procrastination (Waldron et al. 2018).

Because the program also emphasized underlying motivations for completing tasks, it likely fostered greater intrinsic motivation and goal commitment. Intrinsic motivation supports goal‐setting and sustained effort, and increasing motivational readiness may enhance engagement in academic responsibilities (Visser et al. 2020). Taken together, these mechanisms align with the view that procrastination can reflect short‐term mood management; interventions that increase tolerance of discomfort, reduce avoidance, and strengthen self‐regulation are therefore well positioned to reduce procrastination (Sirois 2023).

Psychotherapists working with students or clients in high‐pressure environments should be attuned to early signs of procrastination and its emotional underpinnings. Early intervention can prevent procrastination from becoming entrenched, thereby mitigating its negative impact on academic performance and overall well‐being. The intervention package developed in this study suggests that procrastination is a multifaceted behavior influenced by emotional, cognitive, and situational factors. Psychotherapists should avoid a one‐size‐fits‐all approach and instead tailor interventions to the specific causes and manifestations of procrastination in each client. Psychoeducational components that explain the psychological roots and consequences of procrastination may increase insight, reduce shame, and motivate change. Additionally, given the salience of cyberspace‐related distraction in our model, clinicians may consider incorporating structured strategies for digital self‐management and reinforcement scheduling alongside cognitive and emotion regulation skills (Kaliba and Ambrožová 2021; Steel 2007).

Several limitations should be considered when interpreting these findings. First, the qualitative phase relied on a relatively small interview sample drawn from a single university context, which may limit the transferability of the grounded theory model to other educational settings or cultures. Second, the quantitative phase used a quasi‐experimental pre‐test/post‐test control‐group design; although random assignment to groups was conducted, unmeasured contextual or individual confounds may still influence outcomes. Third, procrastination was assessed via self‐report, which can be affected by response bias and may not fully capture behavioral indicators such as objective assignment submission patterns. Fourth, outcomes were evaluated at post‐test; future studies should include follow‐up assessments to test durability of effects and potential relapse. Finally, because the intervention package is multi‐component, dismantling designs or mediation analyses are recommended to identify which elements (e.g., cognitive restructuring, mindfulness skills, motivational enhancement, time‐management practices) account for change, and for whom.

### 4.2 Conclusion

This mixed‐methods study developed a grounded theory explanation of academic procrastination and translated the resulting model into a structured, mechanism‐informed intervention package. Qualitative findings identified interacting causal, contextual, and mediating conditions that shape coping strategies and lead to educational, psychological, and interpersonal consequences, with a core category reflecting enduring personality, family, and motivational patterns. Quantitative results further supported the effectiveness of the grounded‐theory‐derived package in reducing academic procrastination. Collectively, these findings suggest that interventions targeting cognitive distortions, emotional regulation, motivational states, and contextual distractions may offer an effective and practical pathway for reducing academic procrastination among students. Future research should test generalizability across settings, include longer‐term follow‐up, and clarify active mechanisms of change through rigorous component and mediation analyses.

## Funding

The authors have nothing to report.

## Ethics Statement

The study was conducted in strict compliance with ethical principles. The Ethics Review Board of Bushehr University of Medical Sciences approved the present study with the code of IR.BPUMS.REC.1403.151. Also, written informed consent was obtained from the participants.

## Consent

Informed consent was obtained from all participants prior to study commencement.

## Conflicts of Interest

The authors declare no conflicts of interest.

## Supporting information


**Appendix A:** Semi‐structured interview guide (Study 1).

## Data Availability

The data that support the findings of this study are available on request from the corresponding author. The data are not publicly available due to privacy or ethical restrictions.
