# Supplementary material for: Design, Development and Validation of a Training Package for Treating Procrastination in Students Based on Grounded Theory: A Mixed‐Methods Approach
Source: Psych J. 2026 Jul 1;15(4):e70114. doi: 10.1002/pchj.70114 (PMC13322781; doi:10.1002/pchj.70114)
Supplement: Supplementary file 1 — Appendix A: Semi‐structured interview guide (Study 1). [file PCHJ-15-e70114-s001.docx]

**Appendix A. Semi-Structured Interview Guide (Study 1)**

### A1. Opening and rapport

1. Please tell me a little about yourself (major/year of study) and what a typical study week looks like for you.
2. When you hear the term “academic procrastination,” what does it mean to you in your own experience?

### A2. Causal factors (RQ1)

1. Think of a recent time you delayed an important academic task. What do you think caused the delay?
   **Probes:** fear of failure, perfectionism, low self-confidence, lack of motivation, fatigue/sleep, mood, workload.
2. Are there particular types of tasks you postpone more than others? Why?

### A3. Contextual and mediating factors (RQ2)

1. What conditions in your environment make procrastination more likely?
   **Probes:** family climate/support/supervision, dormitory life, class quality, academic pressure, peer influence.
2. How does cyberspace (phone/social media/internet) affect your procrastination? Can you give an example?
3. Did the COVID period/online classes change your procrastination patterns? How?

### A4. Actions/strategies (RQ3)

1. What do you usually do when you notice you are procrastinating?
   **Probes:** avoidance (doing unrelated things), turning to cyberspace, sleeping, planning/notes, self-motivation, seeking help.
2. What strategies have you tried to reduce procrastination? Which ones worked, and which did not?

### A5. Consequences (RQ4)

1. What happens academically when you procrastinate?
   **Probes:** grades, missed deadlines, absences, dropping courses.
2. What emotional or psychological consequences do you experience?
   **Probes:** stress, anxiety, guilt, regret, self-criticism.
3. How does procrastination affect your relationships or how others see you?
   **Probes:** conflict, loss of trust, irritability/aggression.

### A6. Core phenomenon and meaning-making (RQ5)

1. If you had to describe the “core reason” procrastination continues for you, what would it be?
2. In your view, what would need to change for procrastination to reduce in a lasting way?

### A7. Closing

1. Is there anything important about your procrastination experience that we didn’t ask but you think matters?
2. Do you have any suggestions for a training program that could genuinely help students like you?

**Field notes / interviewer memos (recommended to mention briefly):**

- Record contextual observations (setting, notable emotional reactions).
- Note emerging categories to inform theoretical sampling in subsequent interviews.
